# New Insights into the Role of Oxidative Stress in Onset of Cardiovascular Disease

**DOI:** 10.1155/2018/9563831

**Published:** 2018-04-15

**Authors:** Adrian Doroszko, Piotr Dobrowolski, Aneta Radziwon-Balicka, Robert Skomro

**Affiliations:** ^1^Department of Internal Medicine, Hypertension and Clinical Oncology, Wroclaw Medical University, Wroclaw, Poland; ^2^Department of Congenital Heart Diseases, Institute of Cardiology, Warsaw, Poland; ^3^Department of Clinical Experimental Research, Glostrup Research Institute, Rigshospitalet, Glostrup, Denmark; ^4^Division of Respirology, Critical Care and Sleep Medicine, Department of Medicine, University of Saskatchewan, Saskatoon, SK, Canada

Cardiovascular disease is one of the major healthcare problems of the world population. Understanding its determinants is essential for designing effective therapeutic interventions. Cardiorespiratory failure is still among the most common reasons of mortality and morbidity. Since advances in molecular medicine have enabled us to identify the crucial regulatory mechanisms of pathophysiological pathways, it is now possible to develop novel therapeutic strategies, based on evidence from molecular studies.

Numerous studies have shown that endothelium plays a pivotal role in the maintenance of appropriate vascular tone and structure and all the disturbances initiating the onset and promoting the progression of atherosclerosis come from these cells. Vascular endothelium is critically located between blood and the vascular wall and is among the largest endocrine organ in terms of surface (400–4000 m^2^), its mass reaching 1.5 kg. Under physiological condition, by releasing numerous mediators of auto- and paracrine action, it performs the anti-inflammatory, antiaggregatory, vasodilative, and antiproliferative functions.

All cells are capable of producing reactive oxygen species (ROS), and some evidence suggests that ROS produced by cardiac myocytes and vascular smooth muscle cells play an important role in the development and progression of cardiovascular disease. Endothelial dysfunction is characterized by decreased vasodilative potential as well as increased inflammatory and aggregatory activity. The mechanisms underlying decreased vasodilative action include decreased nitric oxide (NO) bioavailability, oxidative stress, changes in the arachidonic acid metabolite biotransformation, and activation of the renin-angiotensin-aldosterone system (RAAS). All the pathological mechanisms lead to increased platelet aggregation, inflammatory reaction in the vascular wall promoting development of atherosclerosis and its consequences. Endothelial activation under pathological condition is associated with increased mortality in numerous diseases.

Since the platelets are among the most important determinants of pathological modifications of vascular repair, the use of functional proteomics should enable the assessment of the factors affecting an individual platelet functional variability. In addition, a paracrine effect of endothelium on platelet function is an important issue. It is well known that activation of endothelium activates platelets and that antiplatelet drugs improve endothelial function—for instance, platelet aggregation may be inhibited by nitric oxide (NO). The platelet NO concentration is low and it seems that no expression of the nitric oxide synthase in platelets is present. However, the effect of NO on platelets is mediated through the activation of the platelet guanylyl cyclase and production of cGMP.

This special issue is aimed at stimulating the continuing effort to understand the molecular mechanisms of cardiovascular damage induced or mediated by oxidative stress.

M. Jakubowski et al. in a proteomic LC/MS study analysed the molecular mechanism underlying aspirin resistance. Interestingly, the authors found that carbonic anhydrase II, a forgotten enzyme, described for the first time over 30 years ago, was the only discriminatory protein affecting aspirin responsiveness. Increased activity and/or concentration of CA II in platelets should be rated as a new independent risk factor for aspirin resistance and thus for thromboembolic events. Since numerous carbonic anhydrase inhibitors are already well known, and registered for use in humans, the authors postulate to use these drugs in clinical setting, especially in patients with increased platelet activity/amount of carbonic anhydrase. Among the CA II-dependent mechanisms modifying platelet responsiveness, the pH changes of platelet cytosol leading to impaired acetylating of cyclooxygenase by ASA are noteworthy. This in turn could affect the antiplatelet effect of ASA as well as platelet inflammatory activity and energetic metabolism. The CA II activity in platelets has been hypothesized since the proton efflux following platelet thrombin stimulation was described. This was followed by demonstration of the presence of CO_2_ hydration in platelets, which was inhibited by ethoxzolamide, a carbonic anhydrase II inhibitor. Interestingly, carbonic anhydrase II was found to catalyze generation of nitric oxide form nitrite, which may be an additional origination of intraplatelet NO. This reaction is significantly enhanced by both decreased pH and dorzolamide, which are inhibitors of CAII main activity.

Peroxynitrite (ONOO^−^) is a highly reactive oxidant which is generated from the coupling between the endothelial nitric oxide and superoxide. Its detrimental action on the development of cardiac injury as well as its negative effects on cardiac systolic function has been well established. P. Rola et al. intended to investigate if nitric oxide, a potent antiplatelet factor, could be a potential transmitter of the low-level laser therapy-induced modification of platelet activity. In order to explore the impact of the low-level laser therapy on platelet activation, the levels of the platelet factor 4 (PF-4) and sP-selectin were measured both at baseline and following the laser irradiation. The authors have demonstrated that the low level laser therapy decreases the whole-blood platelet aggregation regardless of the NO bioavailability or changes in the platelet activation markers.

J. Sun and colleagues in a review paper indicate that endothelial dysfunction and endothelial nitric oxide synthase (eNOS) dramatically increase the rate of abdominal aortic aneurism (AAA) formation in animal models, pointing at the involvement of endothelium in AAA pathogenesis as well as at possible pharmacological endothelium-related targets in limiting the onset of this disorder. Some authors postulate that AAA and atherosclerosis constitute two separate but related diseases, based on their different clinical patterns. Since there is a paradoxical differential correlation between diabetes and aneurysm formation in the retinal capillaries and the aorta, the authors postulate that deciphering the significance of such a difference could provide better therapeutic strategies for AAA management.

K. Kikuchi et al. examined the effect of edaravone (a reactive oxygen species scavenger) on the thrombolytic effectiveness of alteplase by measuring thrombolysis using a microchip-based flow chamber assay. The thrombolytic effect of alteplase was significantly attenuated in the presence of hydrogen peroxide, suggesting that oxidative stress may limit the thrombolysis. Edaravone alone did not influence thrombosis nor thrombolysis but enhanced alteplase-mediated thrombolysis in vitro, likely by acting as an antioxidant to prevent free radical-related inhibition of alteplase activity on thrombi. Furthermore, edaravone significantly attenuated inhibition of alteplase-induced fibrinolysis by hydrogen peroxide. It would be very interesting to analyse these findings with respect to the risk for severe bleedings related to thrombolytic therapy.

In a review paper, J. Zhang and colleagues discuss how temporal dynamics and localization of activated AMPK and PKA enzymes play a pathogenic role, that is, in diabetes, and propose therapeutic strategies aiming at localized PKA and AMPK signalling to reverse mitochondrial dysfunction, oxidative stress, and death of cardiac and endothelial cells during ischemia and diabetes.

On the other hand, X. Gu et al. reported that acetaldehyde dehydrogenase 2 (ALDH2) is expressed in cardiac fibroblasts and that high glucose concentration may increase oxidative stress-mediated reaction, decrease ALDH2 activity and expression, and induce cardiac fibroblast apoptosis and fibrosis. Activation of ALDH2 may be a defence mechanism by ameliorating the high glucose-induced cardiac fibroblast fibrosis by decreasing oxidative stress and apoptosis.

A. Stanek et al. in a research study demonstrate that increased oxidative stress as well as higher serum concentrations of placental growth factor—PlGF and sCD40L—and increased intima media thickness in carotid arteries may reflect the acceleration of atherosclerosis in male patients in the active phase of ankylosing spondylitis and without concomitant classical cardiovascular risk factors. The authors observed the increased concentration of lipid peroxidation products (malonyldialdehyde (MDA)) in plasma and erythrocytes which could play an important role in LDL modification and their deviation towards macrophages.

To summarize, the manuscripts published in this special issue present recent developments in assessment of cardiovascular consequences of oxidative stress including modulation in platelet aggregation, coagulation, or fibrinolysis. Importantly, these papers also unmask many challenging issues and present novel therapeutic approaches. All articles involved in this special issue had brought about new and valuable information on the role of oxidative stress in the development of cardiovascular disease. We believe that some of the presented studies will provide new evidence, which could lead to the discovery of potential drug targets for the development of new therapeutic approaches for combating cardiovascular disease in the future.

*Adrian Doroszko* *Piotr Dobrowolski* *Aneta Radziwon-Balicka* *Robert Skomro*

